# JMJD1C Regulates Megakaryopoiesis in In Vitro Models through the Actin Network

**DOI:** 10.3390/cells11223660

**Published:** 2022-11-18

**Authors:** Jialing Wang, Xiaodan Liu, Haixia Wang, Lili Qin, Anhua Feng, Daoxin Qi, Haihua Wang, Yao Zhao, Lihua Kong, Haiying Wang, Lin Wang, Zhenbo Hu, Xin Xu

**Affiliations:** 1 Laboratory for Stem Cell and Regenerative Medicine, the Affiliated Hospital of Weifang Medical University, Weifang 261031, China; 2 School of Life Science and Technology, Weifang Medical University, Weifang 261053, China; 3 Department of Blood Transfusion, the Affiliated Hospital of Weifang Medical University, Weifang 261031, China; 4 Department of Hematology, the Affiliated Hospital of Weifang Medical University, Weifang 261031, China; 5 The School of Physics and Electronic Information, Weifang University, Weifang 261061, China

**Keywords:** JMJD1C, megakaryopoiesis, cytoskeleton, actin, Ran GTPase

## Abstract

The histone demethylase JMJD1C is associated with human platelet counts. The JMJD1C knockout in zebrafish and mice leads to the ablation of megakaryocyte–erythroid lineage anemia. However, the specific expression, function, and mechanism of JMJD1C in megakaryopoiesis remain unknown. Here, we used cell line models, cord blood cells, and thrombocytopenia samples, to detect the JMJD1C expression. ShRNA of JMJD1C and a specific peptide agonist of JMJD1C, SAH-JZ3, were used to explore the JMJD1C function in the cell line models. The actin ratio in megakaryopoiesis for the JMJDC modulation was also measured. Mass spectrometry was used to identify the JMJD1C-interacting proteins. We first show the JMJD1C expression difference in the PMA-induced cell line models, the thrombopoietin (TPO)-induced megakaryocyte differentiation of the cord blood cells, and also the thrombocytopenia patients, compared to the normal controls. The ShRNA of JMJD1C and SAH-JZ3 showed different effects, which were consistent with the expression of JMJD1C in the cell line models. The effort to find the underlying mechanism of JMJD1C in megakaryopoiesis, led to the discovery that SAH-JZ3 decreases F-actin in K562 cells and increases F-actin in MEG-01 cells. We further performed mass spectrometry to identify the potential JMJD1C-interacting proteins and found that the important Ran GTPase interacts with JMJD1C. To sum up, JMJD1C probably regulates megakaryopoiesis by influencing the actin network.

## 1. Introduction

Genetic variants of the histone demethylase JMJD1C have been found to be associated with platelet traits, especially counts [1,2,3,4,5,6,7], as well as the VEGF levels, which are also relevant to platelet the functions [8,9,10], according to a large number of genome-wide association studies (GWAS). However, it remains unknown about how JMJD1C could contribute to the platelet physiology, and a detailed summary of JMJD1C in megakaryopoiesis is lacking.

Megakaryopoiesis is a complicated process that can be classified into several stages. Megakaryopoiesis starts from hematopoietic stem cells, which then differentiate into mega–erythroid progenitors, megakaryocytes, proplatelets, and platelets. Cytokines are important for megakaryopoiesis. For example, thrombopoietin (TPO) was the first identified main regulator of the platelet formation. In addition, the signaling pathways downstream of the cytokine receptors determine the platelet formation. Moreover, at each developmental stage of megakaryopoiesis, multiple transcription factors are involved. For example, RUNX1 is involved in every stage and NF-E2 is mainly involved in the transitions from megakaryocytes to proplatelets and from proplatelets to platelets [11].

Megakaryopoiesis has been studied using different platforms, including cell line models. Established in 1985, the cell line MEG-01 is the first cell line that has the ability to produce platelet-like particles and is considered to be the most suitable cell line for analyzing human megakaryocytic maturation and differentiation [12,13]. The cell line K562 was established in 1975, representing a bipotent cell line which is capable of differentiating into either megakaryocyte or erythroid [14]. The cell line HEL was established in 1982 and is a cell line capable of spontaneous and induced globin synthesis, representing a system for studying erythroid cell differentiation, but could also be induced to experience the megakaryocytic differentiation [15]. Thus, MEG-01 represents the late stages of megakaryopoiesis, K562 represents the early stage of megakaryopoiesis when megakaryocyte or erythroid starts to branch. MEG-01, K562, and HEL cells experience a megakaryocytic differentiation when treated with phorbol-12-myristate-13-acetate (PMA, also called TPA), a PKC activator.

We tried to explore the correlation between JMJD1C and megakaryopoiesis that may explain the GWAS of JMJD1C and the platelet counts. We first used the cell line models, cord blood cells, and clinical thrombocytopenia samples to study the expression of JMJD1C in megakaryocytopoiesis. We also used the JMJD1C knockdown along with a specific peptide agonist of JMJD1C, to study the function of JMJD1C. To understand the underlying mechanism, we used mass spectrometry to identify the potential JMJD1C-interacting proteins. We found the specific expression of JMJD1C, demonstrated the function of JMJD1C, and also identified potential JMJD1C-interacting proteins in megakaryopoiesis. To sum up, JMJD1C probably regulates megakaryopoiesis, by influencing the actin network.

## 2. Materials and Methods

### 2.1. Reagents, Human Cell Lines, and Primary Samples

JDI-16, a small molecular inhibitor of JMJD1C, that has been described previously, was purchased from Topscience (Shanghai, China) [16]. Stapled peptides were synthesized by ChinaPeptides (Suzhou, China). PMA was purchased from Sigma (Shanghai, China). TPO was purchased from PeproTech (Suzhou, China).

Human cell lines K562, HEL, MEG-01, MV4-11, HL60, Jurkat, SEM, NK-92, MOLM-13, and THP-1 were obtained from the Leibniz-Institute DSMZ-German Collection of Microorganisms and Cell Cultures (Braunschweig, Germany). K562, HEL, MEG-01, HL60, Jurkat, and SEM were cultured using 90% RPMI 1640 medium, supplemented with 10% fetal bovine serum (FBS). MV4-11, MOLM-13, and THP-1 were cultured using 80% RPMI 1640 medium, supplemented with 20% fetal bovine serum. NK-92 was cultured using 75% α-MEM medium, supplemented with 12.5% FBS, 12.5% horse serum, and 30 U/mL IL2. All cells were incubated in 5% CO_2_ atmosphere at 37 °C. All cells were authenticated using the standard STR (short tandem repeats) genotyping method (ANSI/ATCC ASN-0002-2011) [17].

The primary samples from thrombocytopenia patients and umbilical cord blood cells of healthy newborns were provided by the Affiliated Hospital of Weifang Medical University and Weifang People’s Hospital. Peripheral blood mononuclear cells were collected from thrombocytopenia patients and healthy umbilical cord blood cells, using a Ficoll method, immediately after the samples were obtained. The in vitro culture of megakaryocytes has been reported previously [18]. In particular, CD34^+^ cells were obtained and isolated through a positive selection using an immunomagnetic bead cell-sorting system (Miltenyi Biotec, Shanghai, China) and cultured in serum-free medium in the presence of recombinant human TPO (rhTPO; 10 ng/mL; PeproTech, Suzhou, China). 

### 2.2. Design of the Stabilized Alpha Helix of the JMJD1C Peptides

SAH (stabilized alpha helix)-JMJD1C peptides were designed, based on the zinc finger and jumonji domains of JMJD1C, with the replacement of unnatural amino acids (X; X = (S)-4-pentenylalanine) with olefinic residues crosslinked.

### 2.3. Peptide Incubation

The indicated cell lines were cultured, as shown above, and treated with peptides for 2–6 days. A total of 0.25 million cells were seeded, and immediately after seeding, the cells were treated with peptides for other days, as indicated. A 4-h treatment of the peptides without serum was performed prior to incubation. Cells were then collected for the downstream applications.

### 2.4. Transfection

The cell lines were transfected using shRNA (TCCACCTCCAGAGACTATAAA, shRNA-2; GAGATGTGGAGACCTAATAAT, shRNA-3) against JMJD1C. A scramble shRNA control from *Caenorhabditis elegans,* that does not recognize any human sequence, was used. For the lentivirus transfection, an MOI (multiplicity of infection) value of 50 was used for K562 and MEG-01. The lentivirus carrying shRNA was obtained from GenePharma (Shanghai, China).

### 2.5. Quantitative PCR (qPCR)

To measure the gene expression, the cells were used for the RNA isolation. Two micrograms of total RNA from each cell type was reverse-transcribed into cDNA, using Invitrogen Superscript II Reverse Transcriptase (Life Technologies, Shanghai, China), according to the manufacturer’s instructions. Random primers were used to obtain cDNA. The synthesized cDNA served as the template in the 20 µL qPCR reactions. qPCR was performed using SYBR protocols (Takara, Dalian, China). The PCR was run in an ABI7500 fast real-time PCR machine with qPCR cycling conditions of 95 °C for 30 s, followed by 40 cycles of 95 °C for 3 s and 60 °C for 30 s. The relative concentrations of each target template were calculated according to the comparative Ct method. The expression of the target transcripts was standardized to GAPDH. The qPCR analyses were performed in triplicate. The mean Ct value and standard deviations for each figure were shown in Supplementary Table S1. The primers used were as follows: JMJD1C, forward, CGACGCAGGTCTCGTGCCAA, reverse, TGGGCACGTGTATAATGGCTGTGA; GAPDH, forward, TGGGTGTGAACCATGAGAAGT, reverse, TGAGTCCTTCCACGATACCAA; CD41, forward, GATGAGACCCGAAATGTAGGC, reverse, GTCTTTTCTAGGACGTTCCAGTG; CD61, forward, GTGACCTGAAGGAGAATCTGC, reverse, CCGGAGTGCAATCCTCTGG.

### 2.6. Western Blotting

The total cellular proteins were run on 7.5–15% SDS-PAGE gels for electrophoresis. Anti-JMJD1C (17-10262, Sigma, Shanghai, China), anti-Ran (ab155103, Abcam, Shanghai, China), anti-histone H3 (#06-755, Millipore, Darmstadt, Germany), anti-H3K9-me1 (#07-450, Millipore, Darmstadt, Germany), anti-H3K9-me2 (#07-441, Millipore, Darmstadt, Germany), anti-SCD (sc-58420, Santa Cruz Biotechnology, Shanghai, China), anti-KDM3B (3100s, Cell Signaling Technology, Shanghai, China), and anti-GAPDH (10494-1-AP, Proteintech, Wuhan, China) antibodies were used for the primary detection. As the secondary antibody, either anti-rabbit or anti-mouse IgG, conjugated with horseradish peroxidase (HRP) (GE Healthcare, Braunschweig, Germany) was used. Western Lightning Plus ECL (Perkin Elmer, Waltham, MA, USA) reagents were used for the fluorescence production, and AI600 (GE Healthcare, Shanghai, China) was used for the fluorescence detection to visualize the proteins detected.

### 2.7. Wright–Giemsa Staining 

K562, HEL, and MEG-01 cells were incubated with PMA for 72 h and then collected. The slides were created using a cytospin and subsequently air-dried. The cells were stained with Wright–Giemsa and observed for morphological features using a light microscope. 

### 2.8. Flow Cytometry 

K562 and MEG-01 cells were treated with or without SAH-JZ3 (10 μM), accompanied by 1 or 10 nM PMA for 72h, respectively. The cells were collected, washed using PBS, and stained with CD41-FITC (555466, BD Biosciences, Shanghai, China) and PE Mouse Anti-Human CD61(555754, BD Biosciences), for the detection of CD41 ^+^ CD61^+^ cells. Single positive staining was used to establish the gates. The cell size of the cells being generated were checked and the cell debris in the background was ruled out. The experiments were performed in triple replicates. Flow cytometry was performed and analyzed using DxFlex (Beckman Coulter, Atlanta, GA, USA).

### 2.9. Cell Proliferation Assay 

The cells were plated at the optimal seeding density, 24 h before treatment (in triplicate) with peptides or 0.1% DMSO. The plates were incubated for up to 6 days at 37 °C in 5% CO2. The cells were then lysed with a ViaLight Plus kit (Lonza, Cologne, Germany), according to the manufacturer’s protocol, and the chemiluminescent signal was detected with a TECAN Spark 10M microplate reader (Crailsheim, Germany). 

### 2.10. Assessment of the Actin Polymerization

The actin polymerization assessment has been reported previously [19]. In particular, the levels of the polymerized (F-actin) and depolymerized (G-actin) forms of actin were measured. Equal numbers (0.5 × 10^6^) of cells were lysed in actin-stabilizing buffer (0.1 M PIPES (piperazine-N,N0-bis{2-ethane-sulfonic acid}), 30% glycerol, 5% DMSO, 1 mM MgSO4, 1 mM EGTA, 1% Triton X-100,1 mM ATP, and 40 mg/mL protease inhibitor cocktail). The cells were dislodged by scraping, and the entire extract was centrifuged at 4 °C for 75 min at 16,000× *g*. The supernatant containing G-actin was separated, and the pellet containing F-actin was solubilized with an actin depolymerization buffer (0.1 M PIPES pH = 6.9, 1 mM MgSO4, 10 mM CaCl2, 5 μM Cytochalasin D). The supernatant and the pellets were resuspended separately in a protein lysis buffer and analyzed by western blotting with β-actin antibody (20536, Proteintech, Beijing, China).

### 2.11. Mass Spectrometry

Cord blood cells were isolated and lysed using a non-denaturing lysis buffer (P0013, Biyuntian, Beijing, China). The whole-cell extracts were incubated with two different JMJD1C antibodies (Millipore Cat# 17-10262, RRID: AB_11205409 and Santa Cruz sc-101073, RRID: AB_2234048) and an IgG control. Immunoprecipitation (IP) was performed using protein A/G magnetic beads (Sigma, Shanghai, China). The eluted proteins were dissolved in SDT buffer (4% SDS, 100 mM DTT, 100 mM Tris, protease inhibitor cocktail), and the samples were sent to the Applied Protein Technology (Shanghai, China) for mass spectrometry.

### 2.12. Immunoprecipitation

The cells were lysed using a non-denaturing lysis buffer (P0013, Biyuntian, Beijing, China). Immunoprecipitation (IP) was performed using protein A/G magnetic beads (Sigma, Shanghai, China). The IP-grade antibodies, including anti-JMJD1C (17-10262, Sigma, Shanghai) and anti-Ran (ab155103, Abcam, Shanghai, China) were used to enrich the proteins of interest. Rabbit IgG antibody was used as a control.

### 2.13. Statistical Analysis 

The quantitative results are reported as the mean ± standard deviation (SD). The statistical analysis of the qPCR and the colony units was performed using Student’s *t*-test. The statistical analysis of the JMJD1C expression in the clinical thrombocytopenia samples was performed using the Mann–Whitney U test. Comparisons with a *p* value ≤ 0.05 were considered statistically significant.

## 3. Results

### 3.1. Expression of JMJD1C in Megakaryopoiesis and Thrombocytopenia Patients

**Figure 1:**
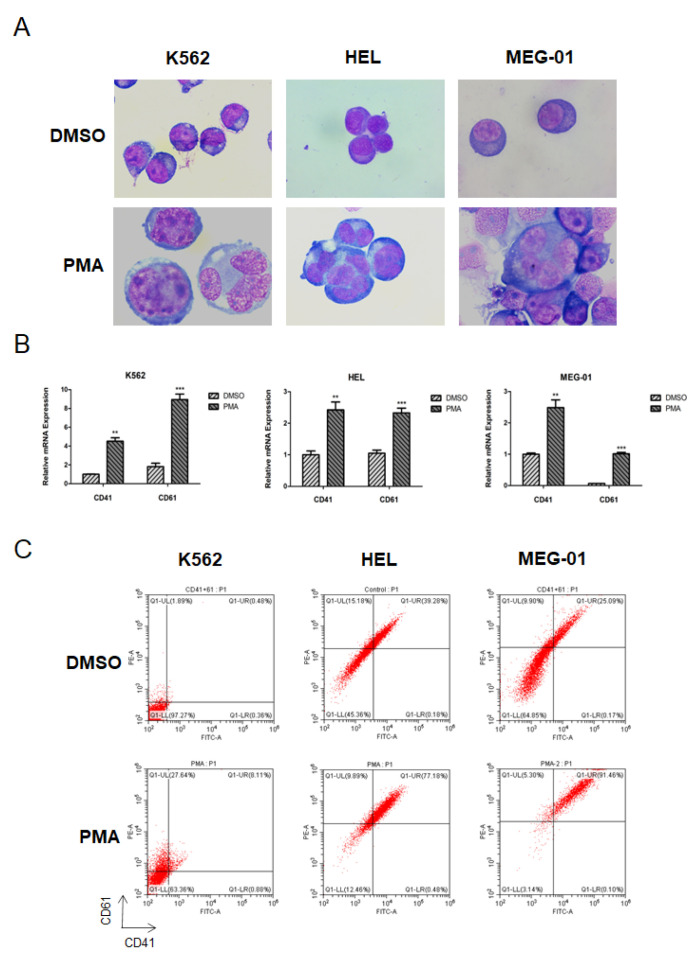
PMA-induced differentiation of K562, HEL, and MEG-01 cells. (**A**) Morphology changes of K562, HEL, and MEG-01 cells upon the PMA treatment. Cells were seeded at 2.5 × 10^5^/mL cells and treated with 1 (K562) or 10 (HEL and MEG-01) nM PMA for 72 h. Cells were then stained with Wright–Giemsa and observed for morphological features, using a light microscope. (**B**) Measurement of the cell differentiation of K562, HEL, and MEG-01 cells upon the PMA treatment by the cell surface markers CD41 and CD61, using the qPCR. Cells were seeded at 2.5 × 10^5^/mL cells and treated with 1 (K562) or 10 (HEL and MEG-01) nM PMA for 72 h. Cells were then collected and lysed in TRIzol buffer. qPCR was performed. Three independent experiments were performed, and a *p* value smaller than 0.05 was regarded as statistically significant. (**C**) Measurement of the cell differentiation of K562, HEL, and MEG-01 cells upon the PMA treatment by the cell surface markers CD41 and CD61, using flow cytometry. Cells were seeded at 2.5 × 10^5^/mL cells and treated with 1 (K562) or 10 (HEL and MEG-01) nM PMA for 72 h. Cells were then collected, and the flow cytometry was used to measure the CD41 and CD61 expression. **, *p* < 0.01; ***, *p* < 0.001.

**Figure 2:**
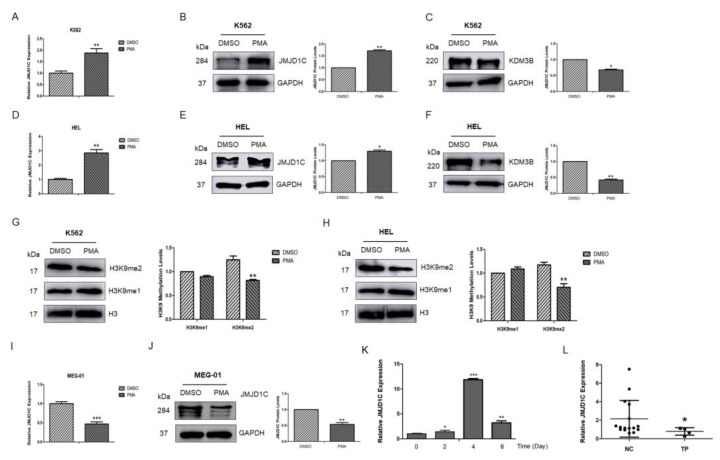
JMJD1C expression in the PMA-induced differentiation of K562, HEL, and MEG-01 cells, TPO-induced differentiation of the cord blood cells, and thrombocytopenia patients. (**A**) JMJD1C mRNA expression in the PMA-induced differentiation of K562 cells. Cells were seeded at 2.5 × 10^5^/mL cells and treated with 1 nM PMA for 72 h. Cells were then collected and lysed in TRIzol buffer. qPCR was performed. Three independent experiments were performed, and a *p* value smaller than 0.05 was regarded as statistically significant. (**B**,**C**) JMJD1C and KDM3B protein expressions in the PMA-induced differentiation of K562 cells. Cells were seeded at 2.5 × 10^5^/mL cells and treated with 1 nM PMA for 72 h. Cells were then collected and lysed in a protein lysis buffer. WB was performed. Three independent experiments were performed. (**D**) Histone 3 lysine 9 mono- and di-methylation (H3K9-me1 and H3K9-me2) levels in the PMA-induced differentiation of K562 cells. Cells were treated, as described above. (**E**) JMJD1C mRNA expression in the PMA (10 nM)-induced differentiation of HEL cells. Cells were treated, as described above. (**F**,**G**) JMJD1C and KDM3B protein expression in the PMA (10 nM)-induced differentiation of HEL cells. Cells were treated, as described above. (**H**) H3K9-me1 and H3K9-me2 levels in the PMA-induced differentiation of HEL cells. Cells were treated, as described above. (**I**) JMJD1C mRNA expression in the PMA (10 nM)-induced differentiation of MEG-01 cells. Cells were treated, as described above. (**J**) JMJD1C protein expression in the PMA (10 nM)-induced differentiation of MEG-01 cells. Cells were treated, as described above. (**K**) JMJD1C mRNA expression in the TPO-induced differentiation of the CD34^+^ human cord blood cells. Human cord blood cells were obtained and CD34^+^ cells were isolated, as described in the Materials and Methods. An amount of 10 ng/mL was used to induce the differentiation of the CD34^+^ human cord blood cells, and the cells were then collected and lysed in TRIzol buffer. qPCR was performed. Three independent experiments were performed, and a *p* value smaller than 0.05 was regarded as statistically significant. (**L**) JMJD1C mRNA levels were measured in peripheral blood mononuclear cells (PBMCs) from 16 healthy subjects and four age-, race-, and sex-matched subjects with thrombocytopenia (Supplementary Table S2, *p* = 0.049, Mann–Whitney U test). The mean expression of JMJD1C in the normal controls and thrombocytopenia patients was 2.049 and 1.184, respectively. PBMCs were obtained, as described in the Materials and Methods, and the cells were used for the qPCR detection. NC, normal controls; TP, thrombocytopenia. *, *p* < 0.05; **, *p* < 0.01; ***, *p* < 0.001. The intensity ratio of each band was calculated by normalizing the control to 1.

Since very less is known about the expression, function, and mechanism of JMJD1C in megakaryopoiesis, we first aimed to detect the JMJD1C expression during megakaryopoiesis. One paper reported that JMJD1C mRNA increased 4-fold in megakaryocytes from stem cells [2]. We chose three broadly accepted cell line models for megakaryopoiesis: MEG-01, K562, and HEL. We treated K562, HEL, and MEG-01 with PMA and measured the expression of JMJD1C. As shown in Figure 1A–C, the PMA treatment resulted in an increased expression of the megakaryopoiesis markers CD41 and CD61 in K562, HEL, and MEG-01 cells. We next measured the expression of JMJD1C in K562, HEL, and MEG-01 cells treated with PMA. As shown in Figure 2A,B,D,E, JMJD1C showed an increased expression in K562 and HEL cells. By contrast, another KDM3 family member, KDM3B, showed a slightly decreased expression in K562 and HEL cells upon the PMA induction (Figure 2C,F). We also detected histone methylation modifications relevant to JMJD1C and KDM3B in K562 and HEL cells. As shown in Figure 2G,H, in both K562 and HEL cells, histone 3 lysine di-methylation (H3K9-me2), but not H3K9-me1, levels decreased upon the PMA induction. Different from K562 and HEL, MEG-01 represents a model of human megakaryocytic differentiation and maturation, and of the production and release of platelets. JMJD1C showed a decreased expression upon the PMA treatment in MEG-01 cells (Figure 2I,J). To compare what we found in K562 and MEG-01 cells, we induced the differentiation of the CD34+ human cord blood cells, using recombinant TPO. We found that, during the TPO-induced differentiation of the cord blood cells, JMJD1C increased first and then decreased (Figure 2K). The data show that during the in vitro megakaryopoiesis, JMJD1C is induced first and then experiences a reduced expression.

We also checked the JMJD1C expression in thrombocytopenia patients (detailed information of the patients, including platelet counts, is shown in Supplementary Table S2). We collected peripheral blood mononuclear cells from normal controls and thrombocytopenia patients and measured the expression of JMJD1C. As shown in Figure 2L, JMJD1C mRNA was relatively lower in thrombocytopenia patients, compared to the normal controls (mean 2.049 vs. 1.184).

### 3.2. Knockdown of JMJD1C Shows Different Influences on Megakaryopoiesis in the Cell Line Models

**Figure 3:**
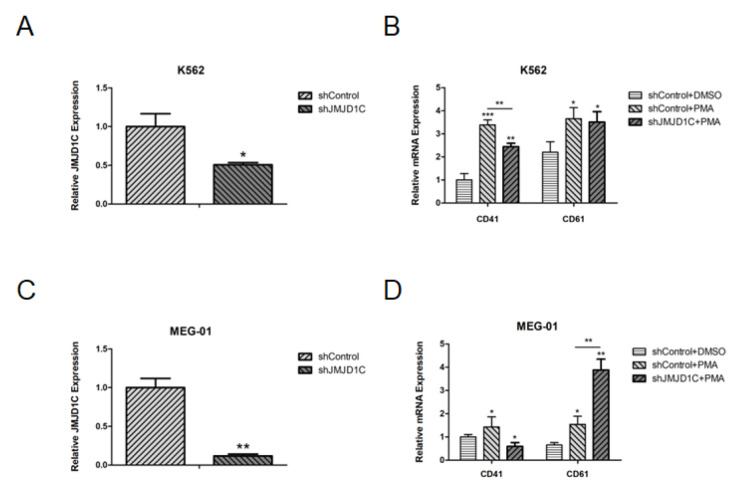
The effect of the JMJD1C knockdown on cell differentiation of K562 and MEG-01 cells. (**A**) JMJD1C knockdown in K562 cells. Cells were seeded at 2.5 × 105/mL cells and infected with lentivirus carrying JMJD1C shRNA for 72 h. Cells were then collected and lysed in TRIzol buffer. qPCR was performed. Three independent experiments were performed, and a *p* value smaller than 0.05 was regarded as statistically significant. (**B**) The effect of JMJD1C on the CD41 and CD61 expression of K562 cells induced by PMA. Cells were seeded at 2.5 × 105/mL cells and treated with 1 nM PMA for 72 h. Cells were then collected and lysed in TRIzol buffer. qPCR was performed. Three independent experiments were performed, and a *p* value smaller than 0.05 was regarded as statistically significant. (**C**) JMJD1C knockdown in MEG-01 cells. Cells were seeded at 2.5 × 105/mL cells and infected with lentivirus carrying JMJD1C shRNA for 72 h. Cells were then collected and lysed in TRIzol buffer. qPCR was performed. Three independent experiments were performed, and a *p* value smaller than 0.05 was regarded as statistically significant. (**D**) The effect of JMJD1C on the CD41 and CD61 expression of MEG-01 cells induced by PMA. Cells were seeded at 2.5 × 105/mL cells and treated with 10 nM PMA for 72 h. Cells were then collected and lysed in TRIzol buffer. qPCR was performed. Three independent experiments were performed, and a *p* value smaller than 0.05 was regarded as statistically significant. *, *p* < 0.05; **, *p* < 0.01; ***, *p* < 0.001.

We then aimed to determine if the JMJD1C knockdown could have any effect on megakaryopoiesis. For this, we performed a knockdown of JMJD1C in K562 and MEG-01 cells. JMJD1C shRNA significantly decreased the JMJD1C expression, as shown in Figure 3A,C. Moreover, the JMJD1C knockdown led to a decreased CD41 expression, induced by PMA in K562 cells (Figure 3B). By contrast, when we performed the knockdown of JMJD1C in MEG-01 cells, we found that the JMJD1C knockdown led to an increased CD61 expression, induced by PMA in MEG-01 cells (Figure 3D). The data show that JMJD1C shows different influences on the PMA-induced differentiation of megakaryopoiesis, depending on the induced JMJD1C expression.

### 3.3. Identification of a Stapled Peptide as a JMJD1C Agonist

**Figure 4:**
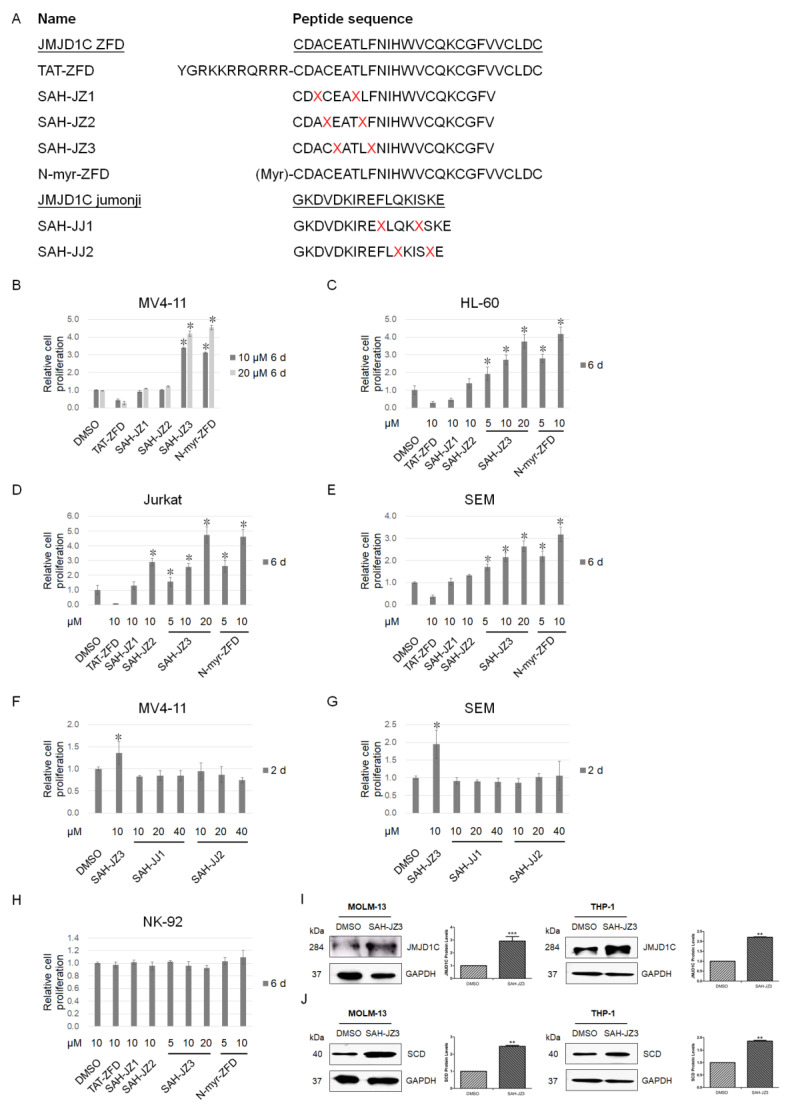
Identification of a peptide agonist of JMJD1C. (**A**) Stapled peptides of the JMJD1C zinc finger and jumonji domains. α,α-Disubstituted non-natural amino acids (X) containing olefinic side chains of varying lengths were synthesized. Non-natural amino acid substitutions were made to flank three (substitution positions i and i + 4) amino acids within the JMJD1C zinc finger and jumonji domains, so that the reactive olefinic residues would reside on the same face of the α-helix. ZFD, zinc finger domain of JMJD1C; TAT, HIV TAT sequence; X = S4; N-myr, myristoylation at the N-terminal of the peptide. (**B**–**H**) The effect of the JMJD1C-originated peptides on the cell proliferation. Cell lines MV4-11, SEM, HL-60, Jurkat, and NK-92 were maintained, as described in the Materials and Methods. Cells were seeded at 30,000/mL in 100 μL medium in V-bottom 96-well plates and incubated with the indicated peptides for the duration shown. Cells were measured for the cell proliferation using an ATP detection kit, as described in the Materials and Methods. Three replicated experiments were performed, and a *p* value smaller than 0.05 was regarded as statistically significant. (**I**,**J**) The effect of the JMJD1C-derived peptide SAH-JZ3 on JMJD1C (**I**) and the SCD (**J**) protein expression. Cell lines MOLM-13 and THP-1 were maintained, as described in the Materials and Methods. Cells were incubated with the indicated peptides for 3 d and then collected for western blotting. Three replicated experiments were performed. *, *p* < 0.05; **, *p* < 0.01; ***, *p* < 0.001. The intensity ratio of each band was calculated by normalizing the control to 1.

To further explore the role of JMJD1C in megakaryopoiesis, we tried to develop JMJD1C modulators. We previously identified small molecular JMJD1C modulators [16,20,21]. We also aimed to identify the peptide modulators of JMJD1C. Considering that stapled peptides have recently been identified to be qualified to increase the peptide stability and permeability [22], we aimed to design stapled peptides of JMJD1C, which usually requires an α-helix structure. A large number of studies have focused on the JMJD1C domains, including the jumonji catalytic domain (aa 2274–2498, NCBI Reference Sequence: NP_116165.1), zinc finger domain (aa 1846–1871, NCBI Reference Sequence: NP_116165.1) [23], androgen receptor (AR) binding domain (aa 1732–1843, GenBank: AAI43723.1), thyroid-hormone receptor (TR) binding domain (aa 1848–1988, GenBank: AAI43723.1) [24], and RNF8/RNF168 interacting domain (aa 753–1162, NCBI Reference Sequence: NP_116165.1) [25]. All of these domains of JMJD1C have been reported to be important for the JMJD1C function. Among them, the jumonji and zinc finger domains of JMJD1C were found by a CRISPR/Cas9-based screening system to be especially critical for leukemia [26]. Moreover, the jumonji domain is responsible for the JMJD1C enzymatic activities, and the zinc finger domain is more conserved than other domains. We thus focused on the jumonji and zinc finger domains of JMJD1C for the peptide design. We scanned the jumonji catalytic and zinc finger domains of JMJD1C, using an online tool for the secondary structure prediction (Jpred 4, http://www.compbio.dundee.ac.uk/jpred/index.html, accessed on 3 May 2021). We selected the i and i + 3 insertions of non-natural amino acids into the JMJD1C zinc finger and jumonji domains (Figure 4A). We also incorporated a JMJD1C zinc finger domain peptide fused with the HIV TAT sequence or modified with N-myristoylation. 

We then treated leukemia cells with these peptides to detect their effect on the cell proliferation. Unexpectedly, multiple leukemia cells, including the *MLL*r acute leukemia cell lines MV4-11 and SEM, AML cell line HL-60, and T-cell leukemia cell line Jurkat, showed an increased proliferation after the treatment with SAH-JZ3, but not the other peptides, as shown in Figure 4B–H. These results indicate that SAH-JZ3 is a peptide that enhances the proliferation of cells of MV4-11 and other cell lines that are dependent on JMJD1C [27,28,29], but not NK-92, which shows a much lower JMJD1C expression (Supplementary Figure S1). We also measured the protein expression of JMJD1C upon the SAH-JZ3 treatment. The SAH-JZ3 incubation can significantly increase JMJD1C itself and its target SCD protein expression in MOLM-13 and THP-1 cells, as shown in Figure 4I,J. These results indicate that SAH-JZ3 is a peptide that increases the JMJD1C protein expression. To sum up, SAH-JZ3 is a potential JMJD1C agonist.

### 3.4. The Peptide Agonist of JMJD1C Shows a Differential Regulation of K562 and MEG-01 Cells

**Figure 5:**
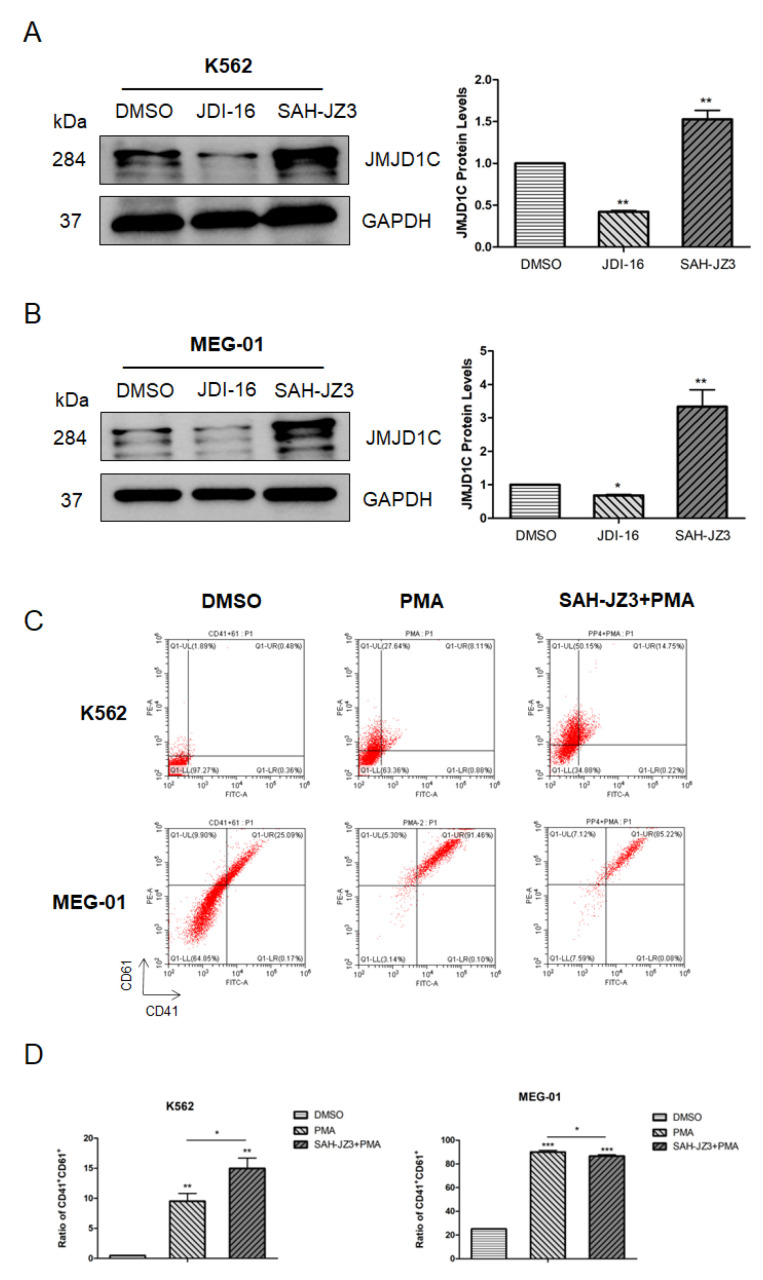
The effect of the peptide agonist of JMJD1C, SAH-JZ3, on the PMA-induced differentiation of K562 and MEG-01 cells. (**A**,**B**) The effect of the small molecular inhibitor of JMJD1C, JDI-16, and the peptide agonist of JMJD1C, SAH-JZ3, on the JMJD1C protein expression in K562 (**A**) and MEG-01 (**B**) cells. Cell lines K562 and MEG-01 were maintained, as described in the Materials and Methods. Cells were incubated with the indicated inhibitor and the peptide for 3 d and then collected for the western blotting. The experiments were performed in triple replicates. (**C**,**D**) The effect of SAH-JZ3 on the PMA-induced cell differentiation of K562 and MEG-01 cells. Cells were seeded at 2.5 × 10^5^/mL cells and treated with 1 (K562) or 10 (MEG-01) nM PMA with or without the addition of SAH-JZ3 (10 μM) for 72 h. Cells were then collected, and the flow cytometry was used to measure the CD41 and CD61 expression. Three replicated experiments were performed. *, *p* < 0.05; **, *p* < 0.01; ***, *p* < 0.001. The intensity ratio of each band was calculated by normalizing the control to 1.

To further explore the role of JMJD1C in megakaryopoiesis, we treated K562 and MEG-01 cells with our identified JMJD1C agonist SAH-JZ3. As shown in Figure 5A,B, SAH-JZ3 also increased the JMJD1C protein expression in these two cell lines. By contrast, the small molecular inhibitor of JMJD1C, JDI-16, did not show an influence on the JMJD1C protein expression in K562 and MEG-01 cells. We further detected the effect of SAH-JZ3 on the PMA-induced differentiation of K562 and MEG-01. As shown in Figure 5C,D, SAH-JZ3 promoted the PMA-induced differentiation of K562 but attenuated the PMA-induced differentiation of MEG-01, consistent with the effect of JMJD1C shRNA, shown in Figure 3. These data demonstrate that the JMJD1C agonist shows a different regulation of the PMA-induced differentiation of K562 and MEG-01 cells.

### 3.5. The Influence of JMJD1C on the Cytoskeleton Network

**Figure 6:**
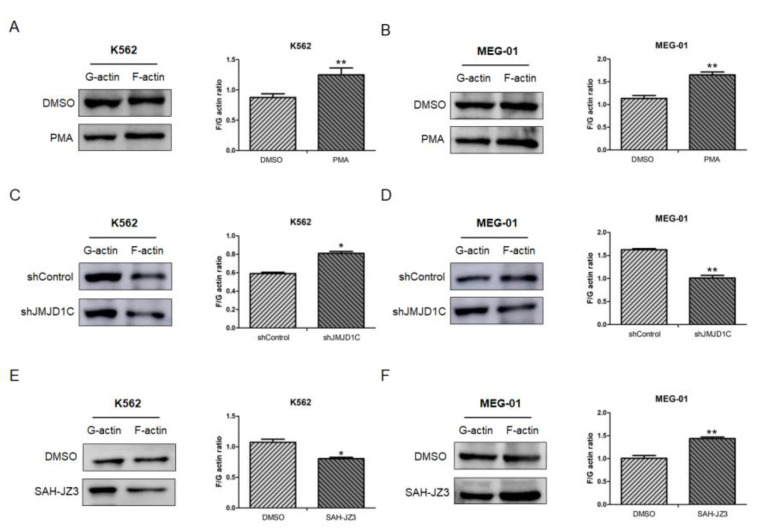
The effect of JMJD1C shRNA and the peptide agonist of JMJD1C, SAH-JZ3, on the actin dynamics of K562 and MEG-01 cells. (**A**,**B**) The effect of PMA on F- and G-actin of K562 (**A**) and MEG-01 (**B**) cells. Cells were seeded at 2.5 × 10^5^/mL cells and treated with PMA (1 nM for K562 and 10 nM for MEG-01) for 72 h. Cells were then collected for the G-actin and F-actin detection, as described in the Materials and Methods. Three replicated experiments were performed. (**C**,**D**) The effect of JMJD1C shRNA on F- and G-actin of K562 (**C**) and MEG-01 (**D**) cells. The experiments were performed, as shown above. (**E**,**F**) The effect of SAH-JZ3 (10 μM) on F- and G-actin of K562 (**E**) and MEG-01 (**F**) cells. The experiments were performed, as shown above. *, *p* < 0.05; **, *p* < 0.01.

Although among all tested traits, the platelet counts demonstrate a strongest association with the JMJD1C genetic variants, according to GWAS, the platelet aggregation is the earliest identified platelet traits with which the JMJD1C genetic variants are associated. Since the cytoskeleton is involved in the platelet aggregation, we next determined the effect of JMJD1C on the cytoskeleton network. The PMA was first used to measure the influence on the actin network. As shown in Figure 6A,B, the PMA treatment resulted in a higher F/G actin ratio in both K562 and MEG-01 cells. We then treated K562 and MEG-01 cells with JMJD1C shRNA. JMJD1C shRNA led to increased and decreased F/G actin ratios in K562 and MEG-01 cells, respectively (Figure 6C,D). By contrast, SAH-JZ3 decreased the F/G actin ratio in K562 and increased the F/G actin ratio in MEG-01 cells (Figure 6E,F). The data show that the JMJD1C agonist SAH-JZ3 increases the F-actin formation in K562 and decreases the actin formation in MEG-01 cells.

### 3.6. JMJD1C Interacts with Ran GTPase

**Figure 7:**
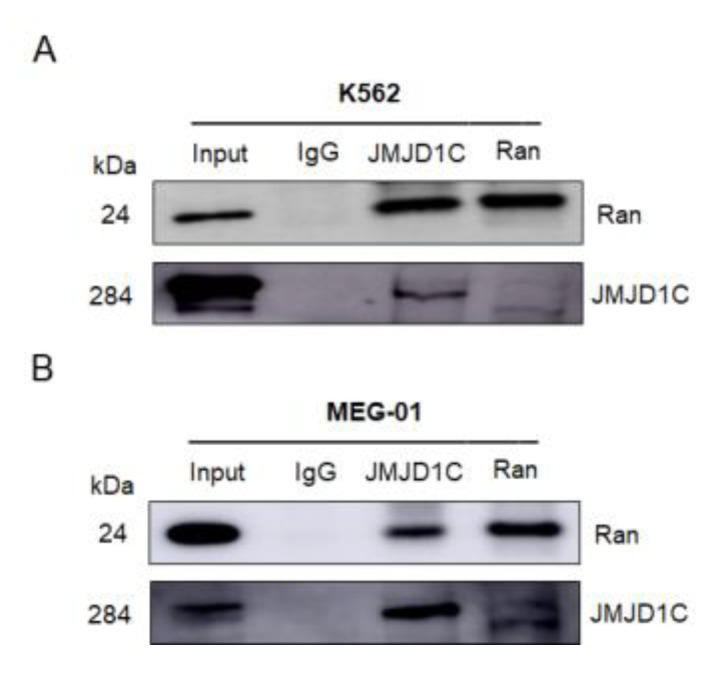
JMJD1C interacts with Ran. (**A**,**B**) Co-immunoprecipitation between JMJD1C and Ran in cell lines K562 (**A**) and MEG-01 (**B**). Cells were lysed in P0013 lysis buffer, and immunoprecipitation was performed using the indicated antibodies. The interacting proteins were detected by western blotting.

We next aimed to identify the interacting proteins of JMJD1C, that may mediate the effect of JMJD1C in the actin network. We first performed mass spectrometry using JMJD1C antibody-immunoprecipitated proteins in cord blood cells. As shown in Supplementary Table S3, in the top 20 potential JMJD1C-interacting proteins identified by the mass spectrometry, numerous actin-regulating proteins were shown. We next tried to confirm the binding of JMJD1C and the identified proteins using immunoprecipitation. Actin, Arp2, Arp3, and IDH2 in the table, along with important actin regulatory proteins, including CDC42, RAC3, MYH9, and CFL1, failed to interact with JMJD1C (Supplementary Figure S2), whereas Ran interacted with JMJD1C in K562 and MEG-01 cells (Figure 7). These data demonstrate that JMJD1C interacts with Ran in K562 and MEG-01 cells.

## 4. Discussion

JMJD1C is found to be related to several platelet traits, especially platelet counts, in GWAS, but the relevant mechanism is unknown. One study showed that the JMJD1C knockdown in zebrafish led to the ablation of the platelet formation [3]. The JMJD1C knockout also resulted in anemia in a mouse model of leukemia [28]. Here, we used megakaryopoiesis cell lines, cord blood cells, and thrombocytopenia samples to explore the role of JMJD1C in megakaryopoiesis. We found that a changed expression of JMJD1C was induced during megakaryopoiesis. The role of JMJD1C on megakaryopoiesis was also explored through the JMJD1C knockdown. We developed a peptide agonist of JMJD1C to study the influence of JMJD1C on megakaryopoiesis. JMJD1C also plays a role in the cytoskeleton network, and JMJD1C is able to interact with an important actin regulator, Ran GTPase. JMJD1C may regulate megakaryopoiesis through the actin network.

Although we focused on the role of JMJD1C in megakaryopoiesis, which is associated with platelet counts, we have to admit that JMJD1C is also probably involved in other platelet traits, including platelet aggregation. In fact, one of the earliest GWAS established the correlation between JMJD1C and platelet aggregation [2]. Later, the same authors reported a further study demonstrating that the strongest association in GWAS is between JMJD1C and the platelet counts [6]. JMJD1C is probably associated with both platelet counts and aggregation since JMJD1C interacts with Ran, which regulates the cytoskeleton network and is important in the platelet aggregation.

Among the known histone demethylases, JMJD1C is probably an important regulator of megakaryopoiesis. JMJD1C, but not its family (KDM3) member KDM3B, shows an association with the platelet traits in GWAS. In our study, the JMJD1C, but not KDM3B, expression was associated with the histone methylation modification changes during megakaryopoiesis (Figure 1). Beyond the KDM3 family, Kitajima et al. reported that a jumonji gene is involved in the regulation of the proliferation, but not differentiation, of the megakaryocyte lineage cells [30]. We analyzed the jumonji cDNA mentioned in the paper, and it turned out to be JARID2, that is structurally similar to the histone demethylase KDM5 family, although it lacks enzymatic activities. Another histone demethylase involved in megakaryopoiesis is PHF2, which is also named KDM7C and functions as a negative epigenetic regulator of the megakaryocyte and erythroid differentiation [31].

We previously identified specific JMJD1C inhibitors that can preferentially kill *MLL*r AML or leukemia stem cells [16,20,21]. Here, we also developed a potential agonist of JMJD1C by adding staples, based on an atypical α-helix in the zinc finger domain of JMJD1C. The zinc finger domain of JMJD1C was chosen because two papers reported that the zinc finger domain is necessary for the enzymatic activities of the JMJD1C jumonji domain [26,32]. Consistent with this, the zinc finger domain is also necessary for the enzymatic activities of KDM1A [33,34]. The zinc finger domain may influence the jumonji domain activities by interacting with other proteins, such as Ran GTPase.

The peptide agonist of JMJD1C can lead to increased JMJD1C proteins. It is possible that the agonist peptide competes for binding to a potential ubiquitin ligase that is responsible for the degradation of JMJD1C. It seems that JMJD1C experiences broad ubiquitin modifications for its stability. For example, potential E3 ubiquitin ligases with the RING finger motif (RNF) RNF8 and RNF168 have been found to show a very robust ubiquitylation of JMJD1C, and more unidentified E3 ubiquitin ligases of JMJD1C are waiting to be identified [25]. Deltex2, another E3 ubiquitin ligase, can also monoubiquitinate JMJD1C, although this is independent of ubiquitylation [35]. It will be interesting to identify ubiquitin ligases that are able to regulate the JMJD1C stability in the platelet formation.

SAH-JZ3 promotes the PMA-induced differentiation of K562 but attenuates the PMA-induced differentiation of MEG-01, probably reflecting the difference between K562 and MEG-01 which represent early and late stages of megakaryopoiesis, respectively. Huang et al. reported that ibrutinib could repress early megakaryopoiesis, but promotes the proplatelet formation [36], supporting that one treatment could lead to dual effects on megakaryopoiesis.

It could be a cell line-specific effect for that SAH-JZ3 decreases F-actin in K562 cells and increases F-actin in MEG-01 cells. The JMJD1C expression is increased and decreased in the PMA-induced K562 and MEG-01 differentiation, respectively. Moreover, during the TPO-induced differentiation of the CD34+ cord blood cells, the JMJD1C expression increased first then decreased with the highest expression at about day 4 of the induction (Figure 2). Interestingly, EZH2, another histone methylation-modulating enzyme, was also induced in early megakaryopoiesis, represented by K562 [37]. Moreover, during the TPO-induced differentiation of the CD34^+^ cord blood cells, the EZH2 expression increased first then decreased with the highest expression at about day 5 of the induction [38]. However, the EZH2 knockdown resulted in the increased actin polymerization in K562 cells, whereas the EZH2 deficiency led to the reduced actin polymerization in the T cells and fibroblast cells [39]. The expression and function of JMJD1C are likely similar to EZH2. As shown in Figure 6C,D, the JMJD1C inhibition by shRNA increased F-actin in K562 but decreased F-actin in MEG-01. SAH-JZ3, as an agonist of JMJD1C that play opposite roles to the JMJD1C inhibition, decreases F-actin in K562 cells and increases F-actin in MEG-01 cells.

Ran GTPase is probably a main effector of JMJD1C in the cytoskeleton regulation. Ran GTPase along with Ras, Rho, Rab, and Arf constitute the Ras superfamily small GTPase which regulates a variety of cell functions. For example, the Rho/CDC42/Rac function as the regulator of the cytoskeleton. Ran is mainly involved in nucleocytoplasmic transport, microtubule organization, and also functions upstream of F-actin [40,41,42]. Furthermore, the Ran expression is higher, compared to Rho/CDC42/Rac in MEG-01 cells [43,44] and is probably the main regulator of the cytoskeleton, among other small GTPase that mediates the effect of JMJD1C on the cytoskeleton network.

There are some limitations in the current study. First, we mainly used cell line systems. Although MEG-01 and K562 are broadly accepted models for megakaryopoiesis, they cannot mimic in vivo biology. Second, we did not assay the proplatelet formation or platelet generation, which is our next aim. Third, the human JMJD1C experiments had a small sample size and were conducted on PBMCs, but not megakaryocytes.

## 5. Conclusions

In conclusion, we summarized the correlation between JMJD1C and megakaryopoiesis and identified a peptide agonist of JMJD1C, as well as the interacting proteins of JMJD1C. It will be interesting to next identify whether JMJD1C contributes to the platelet formation in a way relevant to epigenetics and how JMJD1C is regulated by the ubiquitin-dependent degradation.

## Data Availability

The datasets used during the present study are available from the corresponding authors upon reasonable request.

## Supplementary Materials

The following supporting information can be downloaded at: https://www.mdpi.com/article/10.3390/cells11223660/s1, Table S1, QPCR Ct value for each figure; Table S2: Characteristics of the primary thrombocytopenia samples; Table S3: Potential interacting proteins of JMJD1C from the cord blood cells; Figure S1: JMJD1C expression in MV4-11, Jurkat, and NK-92 cells; Figure S2: Co-immunoprecipitation between JMJD1C and the potential interacting proteins.

## References

[1] Soranzo N., Spector T.D., Mangino M., Kühnel B., Rendon A., Teumer A., Willenborg C., Wright B., Chen L., Li M. (2009). A genome-wide meta-analysis identifies 22 loci associated with eight hematological parameters in the HaemGen consortium. Nat. Genet..

[2] Johnson A.D., Yanek L.R., Chen M.-H., Faraday N., Larson M.G., Tofler G., Lin S.J., Kraja A.T., Province M.A., Yang Q. (2010). Genome-wide meta-analyses identifies seven loci associated with platelet aggregation in response to agonists. Nat. Genet..

[3] Gieger C., Radhakrishnan A., Cvejic A., Tang W., Porcu E., Pistis G., Serbanovic-Canic J., Elling U., Goodall A.H., Labrune Y. (2011). New gene functions in megakaryopoiesis and platelet formation. Nature.

[4] Qayyum R., Snively B.M., Ziv E., Nalls M.A., Liu Y., Tang W., Yanek L.R., Lange L., Evans M.K., Ganesh S. (2012). A Meta-Analysis and Genome-Wide Association Study of Platelet Count and Mean Platelet Volume in African Americans. PLoS Genet..

[5] Wei Y., Wang Z., Su L., Chen F., Tejera P., Bajwa E.K., Wurfel M.M., Lin X., Christiani D.C. (2015). Platelet count mediates the contribution of a genetic variant in LRRC16A to ARDS risk. Chest.

[6] Eicher J.D., Xue L., Ben-Shlomo Y., Beswick A.D., Johnson A.D. (2016). Replication and hematological characterization of human platelet reactivity genetic associations in men from the Caerphilly Prospective Study (CaPS). J. Thromb. Thrombolysis.

[7] Björn N., Sigurgeirsson B., Svedberg A., Pradhananga S., Brandén E., Koyi H., Lewensohn R., de Petris L., Apellániz-Ruiz M., Rodríguez-Antona C. (2020). Genes and variants in hematopoiesis-related pathways are associated with gemcitabine/carboplatin-induced thrombocytopenia. Pharm. J..

[8] Choi S.H., Ruggiero D., Sorice R., Song C., Nutile T., Vernon Smith A., Concas M.P., Traglia M., Barbieri C., Ndiaye N.C. (2016). Six Novel Loci Associated with Circulating VEGF Levels Identified by a Meta-analysis of Genome-Wide Association Studies. PLoS Genet..

[9] Yeung S.L.A., Lam H.S.H.S., Schooling C.M. (2017). Vascular endothelial growth factor and ischemic heart disease risk: A mendelian randomization study. J. Am. Heart Assoc..

[10] Nethander M., Quester J., Vandenput L., Ohlsson C. (2021). Association of Genetically Predicted Serum Estradiol with Risk of Thromboembolism in Men: A Mendelian Randomization Study. J. Clin. Endocrinol. Metab..

[11] Noh J.Y. (2021). Megakaryopoiesis and platelet biology: Roles of transcription factors and emerging clinical implications. Int. J. Mol. Sci..

[12] Ogura M., Morishima Y., Ohno R., Kato Y., Hirabayashi N., Nagura H., Saito H. (1985). Establishment of a novel human megakaryoblastic leukemia cell line, MEG-01, with positive Philadelphia chromosome. Blood.

[13] Takeuchi K., Ogura M., Saito H., Satoh M., Takeuchi M. (1991). Production of platelet-like particles by a human megakaryoblastic leukemia cell line (MEG-01). Exp. Cell Res..

[14] Trinh B.Q., Barengo N., Kim S.B., Lee J.S., Zweidler-McKay P.A., Naora H. (2015). The homeobox gene *DLX4* regulates erythro-megakaryocytic differentiation by stimulating IL-1β and NF-κB signaling. J. Cell Sci..

[15] Martin P., Papayannopoulou T. (1982). HEL cells: A new human erythroleukemia cell line with spontaneous and induced globin expression. Science.

[16] Xu X., Wang L., Hu L., Dirks W.G., Zhao Y., Wei Z., Chen D., Li Z., Wang Z., Han Y. (2019). Small molecular modulators of JMJD1C preferentially inhibit growth of leukemia cells. Int. J. Cancer.

[17] Dirks W., MacLeod R.A., Jäger K., Milch H., Drexler H.G. (1999). First searchable database for DNA profiles of human cell lines: Sequential use of fingerprint techniques for authentication. Cell. Mol. Biol..

[18] Messaoudi K., Ali A., Ishaq R., Palazzo A., Sliwa D., Bluteau O., Souquère S., Muller D., Diop K.M., Rameau P. (2017). Critical role of the HDAC6-cortactin axis in human megakaryocyte maturation leading to a proplatelet-formation defect. Nat. Commun..

[19] Dhenge A., Kuhikar R., Kale V., Limaye L. (2019). Regulation of differentiation of MEG01 to megakaryocytes and platelet-like particles by Valproic acid through Notch3 mediated actin polymerization. Platelets.

[20] Yang Y., Zhang X., Zhang X., Wang Y., Wang X., Hu L., Zhao Y., Wang H., Wang Z., Wang H. (2020). Modulators of histone demethylase JMJD1C selectively target leukemic stem cells. FEBS Open Bio..

[21] Qi D., Wang J., Zhao Y., Yang Y., Wang Y., Wang H., Wang L., Wang Z., Xu X., Hu Z. (2022). JMJD1C-regulated lipid synthesis contributes to the maintenance of MLL-rearranged acute myeloid leukemia. Leuk Lymphoma.

[22] Rezaei Araghi R., Bird G.H., Ryan J.A., Jenson J.M., Godes M., Pritz J.R., Grant R.A., Letai A., Walensky L.D., Keating A.E. (2018). Iterative optimization yields Mcl-1–targeting stapled peptides with selective cytotoxicity to Mcl-1–dependent cancer cells. Proc. Natl. Acad. Sci. USA.

[23] Brauchle M., Yao Z., Arora R., Thigale S., Clay I., Inverardi B., Fletcher J., Taslimi P., Acker M.G., Gerrits B. (2013). Protein Complex Interactor Analysis and Differential Activity of KDM3 Subfamily Members Towards H3K9 Methylation. PLoS ONE.

[24] Wolf S.S., Patchev V.K., Obendorf M. (2007). A novel variant of the putative demethylase gene, s-JMJD1C, is a coactivator of the AR. Arch. Biochem. Biophys..

[25] Watanabe S., Watanabe K., Akimov V., Bartkova J., Blagoev B., Lukas J., Bartek J. (2013). JMJD1C demethylates MDC1 to regulate the RNF8 and BRCA1-mediated chromatin response to DNA breaks. Nat. Struct. Mol. Biol..

[26] Izaguirre-Carbonell J., Christiansen L., Burns R., Schmitz J., Li C., Mokry R.L., Bluemn T., Zheng Y., Shen J., Carlson K.S. (2019). Critical role of Jumonji domain of JMJD1C in MLL-rearranged leukemia. Blood Adv..

[27] Sroczynska P., Cruickshank V.A., Bukowski J.P., Miyagi S., Bagger F.O., Walfridsson J., Schuster M.B., Porse B., Helin K. (2014). ShRNA screening identifies JMJD1C as being required for leukemia maintenance. Blood.

[28] Zhu N., Chen M., Eng R., DeJong J., Sinha A.U., Rahnamay N.F., Koche R., Al-Shahrour F., Minehart J.C., Chen C.-W. (2016). MLL-AF9- and HOXA9-mediated acute myeloid leukemia stem cell self-renewal requires JMJD1C. J. Clin. Investig..

[29] Chen M., Zhu N., Liu X., Laurent B., Tang Z., Eng R., Shi Y., Armstrong S.A., Roeder R.G. (2015). JMJD1C is required for the survival of acute myeloid leukemia by functioning as a coactivator for key transcription factors. Genes Dev..

[30] Kitajima K., Kojima M., Kondo S., Takeuchi T. (2001). A role of jumonji gene in proliferation but not differentiation of megakaryocyte lineage cells. Exp. Hematol..

[31] Yang J., Ma J., Xiong Y., Wang Y., Jin K., Xia W., Chen Q., Huang J., Zhang J., Jiang N. (2018). Epigenetic regulation of megakaryocytic and erythroid differentiation by PHF2 histone demethylase. J Cell Physiol..

[32] Kim S.M., Kim J.Y., Choe N.W., Cho I.H., Kim J.R., Kim D.W., Seol J.E., Lee S.E., Kook H., Nam K.I. (2010). Regulation of mouse steroidogenesis by WHISTLE and JMJD1C through histone methylation balance. Nucleic Acids Res..

[33] Yamane K., Toumazou C., Tsukada Y.I., Erdjument-Bromage H., Tempst P., Wong J., Zhang Y. (2006). JHDM2A, a JmjC-Containing H3K9 Demethylase, Facilitates Transcription Activation by Androgen Receptor. Cell.

[34] Tsukada Y.I., Fang J., Erdjument-Bromage H., Warren M.E., Borchers C.H., Tempst P., Zhang Y. (2006). Histone demethylation by a family of JmjC domain-containing proteins. Nature.

[35] Luo D., de Morree A., Boutet S., Quach N., Natu V., Rustagi A., Rando T.A. (2017). Deltex2 represses MyoD expression and inhibits myogenic differentiation by acting as a negative regulator of Jmjd1c. Proc. Natl. Acad. Sci. USA.

[36] Huang J., Huang S., Ma Z., Lin X., Li X., Huang X., Wang J., Ye W., Li Y., He D. (2021). Ibrutinib Suppresses Early Megakaryopoiesis but Enhances Proplatelet Formation. Thromb. Haemost..

[37] Roy A., Basak N.P., Banerjee S. (2012). Notch1 intracellular domain increases cytoplasmic EZH2 levels during early megakaryopoiesis. Cell Death Dis..

[38] Mazzi S., Dessen P., Vieira M., Dufour V., Cambot M., El Khoury M., Antony-Debré I., Arkoun B., Basso-Valentina F., BenAbdoulahab S. (2019). Dual role of EZH2 in megakaryocyte differentiation. Blood.

[39] Su I.H., Dobenecker M.W., Dickinson E., Oser M., Basavaraj A., Marqueron R., Viale A., Reinberg D., Wülfing C., Tarakhovsky A. (2005). Polycomb group protein ezh2 controls actin polymerization and cell signaling. Cell.

[40] Salus S.S., Demeter J., Sazer S. (2002). The Ran GTPase system in fission yeast affects microtubules and cytokinesis in cells that are competent for nucleocytoplasmic protein transport. Mol. Cell. Biol..

[41] Deng M., Suraneni P., Schultz R.M., Li R. (2006). The Ran GTPase mediates chromatin signaling to control cortical polarity during polar body extrusion in mouse oocytes. Dev. Cell..

[42] Mori M., Yao T., Mishina T., Endoh H., Tanaka M., Yonezawa N., Shimamoto Y., Yonemura S., Yamagata K., Kitajima T.S. (2021). RanGTP and the actin cytoskeleton keep paternal and maternal chromosomes apart during fertilization. J. Cell Biol..

[43] Nagata K., Okano Y., Nozawa Y. (1997). Differential expression of low Mr GTP-binding proteins in human megakaryoblastic leukemia cell line, MEG-01, and their possible involvement in the differentiation process. Thromb. Haemost..

[44] Nagata K., Okano Y., Nozawa Y. (1998). Expression of GTP-binding proteins and protein kinase C isozymes in platelet-like particles derived from megakaryoblastic leukemia cells (MEG-01). Platelets.

