# Supplementary material for: JMJD1C Regulates Megakaryopoiesis in In Vitro Models through the Actin Network
Source: Cells. 2022 Nov 18;11(22):3660. doi: 10.3390/cells11223660 (PMC9688414; doi:10.3390/cells11223660)

Supplementary Materials

JMJD1C Regulates Megakaryopoiesis in in vitro Models through Actin Network

Jialing Wang[1], Xiaodan Liu[1], Haixia Wang[2], Lili Qin[3], Anhua Feng[3], Daoxin Qi[4], Haihua Wang[4], Yao Zhao[4], Lihua Kong[3], Haiying Wang[3], Lin Wang[5], Zhenbo Hu[4*], and Xin Xu[1*]

Contents:

Supplementary Table S1: QPCR Ct value for each figure

Supplementary Table S2: Characteritics of primary thrombocytompenia samples

Supplementary Table S3: Potential interacting proteins of JMJD1C from cord blood cells

Supplementary Figure S1: JMJD1C expression in MV4-11, Jurkat, and NK-92 cells

Supplementary Figure S2: Co-immunoprecipitation between JMJD1C and potential interacting proteins

Supplementary Table S1. QPCR Ct value for each figure.

**Figure 1B**	K562		HEL		MEG-01	
	DMSO	PMA	DMSO	PMA	DMSO	PMA
GAPDH	16.319 ± 0.492	16.678 ± 0.012	16.942 ± 0.049	17.850 ± 0.088	16.908 ± 0.033	17.912 ± 0.029
CD41	29.896 ± 0.061	26.858 ± 0.001	21.926 ± 0.050	21.790 ± 0.125	21.932 ± 0.012	21.621 ± 0.083
CD61	28.835 ± 0.044	24.088 ± 0.080	22.286 ± 0.074	21.956 ± 0.024	25.849 ± 0.003	22.916 ± 0.031

**Figure 2A, D, I**	K562		HEL		MEG-01	
	DMSO	PMA	DMSO	PMA	DMSO	PMA
GAPDH	16.420 ± 0.053	18.013 ± 0.082	16.654 ± 0.030	17.887 ± 0.016	16.908 ± 0.033	17.912 ± 0.029
JMJD1C	24.247 ± 0.068	24.932 ± 0.036	25.886 ± 0.060	25.607 ± 0.070	24.964 ± 0.030	27.055 ± 0.099

**Figure 2K**	Stem Cell			
	0 Day	2 Day	4 Day	6 Day
GAPDH	17.906 ± 0.037	24.631 ± 0.137	27.423 ± 0.168	28.851 ± 0.74
JMJD1C	23.815 ± 0.095	30.019 ± 0.067	31.742 ± 0.149	33.080 ± 0.090

**Figure 2L**	Normal Control			
	#1	#2	#3	#4
GAPDH	23.091 ± 0.406	28.432 ± 0.061	23.421 ± 0.230	27.930 ± 0.132
JMJD1C	25.936 ± 0.003	28.852 ± 0.034	26.062 ± 0.37	28.879 ± 0.049
	#5	#6	#7	#8
GAPDH	24.084 ± 0.024	22.944 ± 0.017	24.272 ± 0.038	22.846 ± 0.064
JMJD1C	26.622 ± 0.025	25.577 ± 0.087	26.654 ± 0.055	25.777 ± 0.054
	#9	#10	#11	#12
GAPDH	24.226 ± 0.026	27.350 ± 0.169	26.230 ± 0.130	23.357 ± 0.104
JMJD1C	24.161 ± 0.057	28.179 ± 0.086	27.949 ± 0.067	26.038 ± 0.043
	#13	#14	#15	#16
GAPDH	23.788 ± 0.005	22.715 ± 0.110	23.104 ± 0.048	23.387 ± 0.051

JMJD1C	26.643 ± 0.058	26.250 ± 0.050	26.545 ± 0.075	25.811 ± 0.027
	Thrombocytopenia			
	#1	#2	#3	#4
GAPDH	20.922 ± 0.086	22.939 ± 0.018	22.543 ± 0.149	22.414 ± 0.036
JMJD1C	26.556 ± 0.205	26.584 ± 0.070	26.140 ± 0.097	26.782 ± 0.052

**Figure**	K562		MEG-01	
**3A, C**	shControl	shJMJD1C	shControl	shJMJD1C
GAPDH	16.533 ± 0.074	16.601 ± 0.029	21.729 ± 0.082	17.900 ± 0.013
JMJD1C	24.050 ± 0.068	24.838 ± 0.097	28.316 ± 0.068	27.575 ± 0.165

**Figure**	K562			MEG-01		
**3B, D**	shControl	shCon+PMA	shJMJ+PMA	shControl	shCon+PMA	shJMJ+PMA
GAPDH	16.532 ± 0.052	16.708 ± 0.018	16.956 ± 0.026	17.815 ± 0.080	23.400 ± 0.136	18.664 ± 0.104
CD41	28.879 ± 0.134	28.014 ± 0.206	28.314 ± 0.035	23.065 ± 0.047	28.132 ± 0.152	24.682 ± 0.222
CD61	28.598 ± 0.062	27.280 ± 0.024	27.716 ± 0.135	23.684 ± 0.113	28.027 ± 0.036	21.960 ± 0.026

Note: Mean Ct value and standard deviations were shown for each figure in the manuscript.

Supplementary Table S2. Characteritics of primary thrombocytompenia samples.

**No.**	**Sex**	**Age**	**WBC (10^9/L, 3.5-9.5)**	**RBC (10^9/L, 4.3-5.8)**	**PLT (10^9/L, 125-350)**	**Classificat ion**
#1	M	79	8.33	5.34	**24**	Moderate
#2	F	79	6.84	4.42	**11**	Severe
#3	F	77	3.95	2.87	**31**	Moderate
#4	M	75	4.29	**3.57**	**30**	Moderate

Note: Characteritics of primary thrombocytompenia samples are summarized. Only thrombocytopenia patients without any other diseases like cancer, bleeding disorders etc. were incorporated. Normal reference range is shown in brackets and values beyond the normal reference range are shown in bold. As for platelet counts, according to the widely accepted international standard, cases are considered mild if platelet counts are between 70 to 150 × 10^9 per L, moderate if platelet counts are between 20 to 70 × 10^9 per L, and severe if platelet counts are less than 20 × 10^9 per L. 16 healthy controls (6 males and 10 femals, mean±SD is 71.8±10.83 years old, by contrast, mean±SD for thrombocytompenia patients are 74.8±6.26) were also recruited for the test and PLTs from these controls are in normal reference range and are not shown. WBC, white blood cells, RBC, red blood cells, PLT, platelets.

Supplementary Table S3. Potential interacting proteins of JMJD1C from cord blood cells.

Identified proteins	Gene name	Protein ID	P value
**ATP synthase subunit alpha, mitochondrial**	ATP5F1A	P25705	1.61E-11
**Profilin-1**	PFN1	P07737	0.0121
**Annexin**	ANXA1	Q5T3N1	0.0005554
**Propionyl-CoA carboxylase beta chain, mitochondrial**	PCCB	E7ETT4	0.0002103
**Actin, cytoplasmic 1**	ACTB	P60709	0.000281
**Actin, cytoplasmic 2**	ACTG	P63261	0.000281
**Myosin light polypeptide 6**	MYL6	F8VZU9	0.02202
**14-3-3 protein beta/alpha**	YWHAB	B5BU24	0.001403
**60S ribosomal protein L13**	RPL13	J3QSB4	0.0004578
**IDP**	IDH2	H0YL11	0.0003685
**40S ribosomal protein S14**	RPS14	E5RH77	0.003648
**GTP-binding nuclear protein Ran**	Ran	P62826	0.01869
**Myeloperoxidase**	MPO	P05164	0.01999
**60S ribosomal protein L27**	RPL27	E4W6B6	0.002125
**Alpha-enolase**	ENO1	P06733	0.04736
**Ras-related protein Rab-21**	Rab21	Q96GX3	0.01951
**Actin-related protein 2**	Arp2	B4DWQ5	0.003878
**Actin-related protein 2/3 complex**	Arp3	B2R4D5	0.0005978

**subunit 3**			
**Histone H1.5**	H1-5	P16401	0.00328
**Phosphoribosylpyro phosphate synthetase isoform I**	PRPS1	Q15244	0.03406

Notes: Mass spectrometry was performed as described in materials and methods. Proteins are ranked according to the score which is calculated by subtracting the ionscore of IgG sample from the ionscore of JMJD1C antibody immunoprecipitated samples.

Supplementary Figure S1. JMJD1C expression in MV4-11, Jurkat, and NK-92 cells. Cell lines were cultured as described in the materials and methods. Cells were collected for protein extraction and Western blot was performed to detect JMJD1C expression in these three cell lines. Experiments were repeated in double replicates.

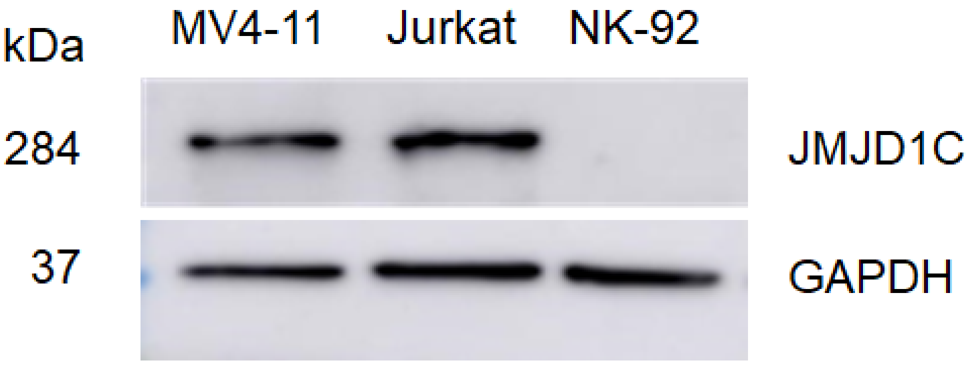

Supplementary Figure S2. Co-immunoprecipitation between JMJD1C and potential interacting proteins. Co-immunoprecipitation between JMJD1C and CDC42, RAC3, Actin, IDH2, Arp2, Arp3, CFL1, and MYH9 was performed as described in the materials and methods.

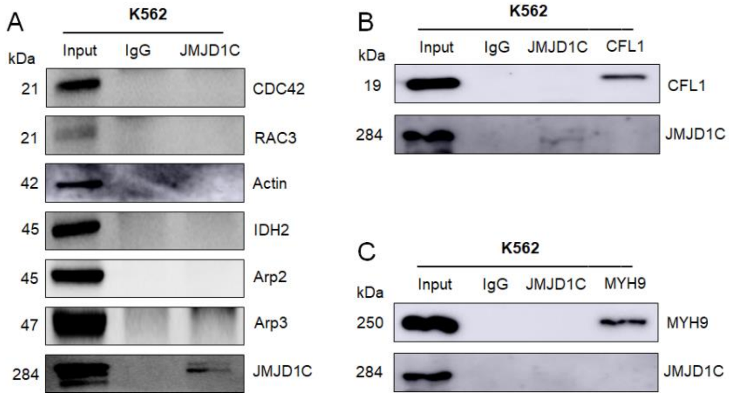

Supplement: Supplementary file 1 [file cells-11-03660-s001.zip › cells-2020795-supplementary.pdf]
